# Novel *TCAP* Mutation c.32C>A Causing Limb Girdle Muscular Dystrophy 2G

**DOI:** 10.1371/journal.pone.0102763

**Published:** 2014-07-23

**Authors:** Amirtharaj Francis, Balaraju Sunitha, Kandavalli Vinodh, Kiran Polavarapu, Shiva Krishna Katkam, Sailesh Modi, M. M. Srinivas Bharath, Narayanappa Gayathri, Atchayaram Nalini, Kumarasamy Thangaraj

**Affiliations:** 1 CSIR-Centre for Cellular and Molecular Biology, Hyderabad, India; 2 Department of Neuropathology, National Institute of Mental Health and Neurosciences, Bengaluru, India; 3 Department of Neurology, National Institute of Mental Health and Neurosciences, Bengaluru, India; 4 Department of Neurochemistry, National Institute of Mental Health and Neurosciences, Bengaluru, India; Stem Cell Research Institute, Belgium

## Abstract

*TCAP* encoded telethonin is a 19 kDa protein, which plays an important role in anchoring titin in Z disc of the sarcomere, and is known to cause LGMD2G, a rare muscle disorder characterised by proximal and distal lower limb weakness, calf hypertrophy and loss of ambulation. A total of 300 individuals with ARLGMD were recruited for this study. Among these we identified 8 clinically well characterised LGMD2G cases from 7 unrelated Dravidian families. Clinical examination revealed predominantly proximo - distal form of weakness, scapular winging, muscle atrophy, calf hypertrophy and foot drop, immunoblot showed either complete absence or severe reduction of telethonin. Genetic analysis revealed a novel nonsense homozygous mutation c.32C>A, p.(Ser11*) in three patients of a consanguineous family and an 8 bp homozygous duplication c.26_33dupAGGTGTCG, p.(Arg12fs31*) in another patient. Both mutations possibly lead to truncated protein or nonsense mediated decay. We could not find any functionally significant *TCAP* mutation in the remaining 6 samples, except for two other polymorphisms, c.453A>C, *p.( = )* and c.-178G>T, which were found in cases and controls. This is the first report from India to demonstrate *TCAP* association with LGMD2G.

## Introduction

The seventh form of Autosomal Recessive Limb-Girdle Muscular Dystrophy (LGMD2G; OMIM 601954) is caused by genetic variations in the telethonin gene (also named as *TCAP* or titin-cap gene) (NM_003673.3). LGMD2G has been mapped to chromosome 17q12 [1]. LGMD2G was first described from Brazil [2]; subsequently it was reported from China [3], Moldavia [4], and Portugal [5]. Onset of this disease is usually in the second decade, with predominant pelvic girdle weakness and associated distal weakness with foot drop [2]. About50% of the cases are known to have calf hypertrophy and 40% are known to lose independent ambulation during the third or fourth decade [1].

Telethonin is amongst the most abundant nuclear mRNA transcribed in skeletal muscle and encodes for a 19 kDa protein consisting of 167 amino acids [6]. The protein is located in the Z-disc of sacromere, the basic functional contractile unit in the skeletal and cardiac muscles [6], [7], [8]. Telethonin plays an important role in the sarcomeric assembly as it joins the two antiparallel titin (Z1-Z2) domains together through beta strand crossing and represents the strongest protein–protein interaction observed so far [9], . Telethonin is also known for its interaction with other Z-disc proteins of sarcomeric assembly such as LIM protein (MLP) [13], Ankrd2 [14], myostatin [15], potassium channel B-subunit minK [16], protein kinase D [17], murine double minute2 (MDM2) [18],and calsarcin [19]. Thus, *TCAP* is expected to play a very important role in the sarcomeric assembly.

It has been demonstrated that the *TCAP* knockout mouse resulted in a modest increase in passive muscle stiffness and shared many of the features of LGMD 2G phenotype [20]. Looking at the functional importance of telethonin in muscles, we have analysed *TCAP* in eight patients of Dravidian origin (south-India) in order to identify *TCAP* mutations in LGMD2G.

## Materials and Methods

### Subjects

This study was approved by the Institutional Ethical Committee (IEC) of Centre for Cellular and Molecular Biology, Hyderabad, India and National Institute of Mental Health and Neurosciences, Bangalore, India. The subject recruitment and sample collection were done after obtaining written informed consent/assent forms from all participants. In this prospective study (February 2010 to September 2012) a total of 300 patients with features of ARLGMD were prospectively evaluated by the principal investigator (AN) at the National Institute of Mental Health and Neurosciences. Of these 300 cases, 246 patients were confirmed to have various forms of ARLGMD based on characteristic clinical phenotype, biochemical and histopathological analysis. IB was performed sequentially based on the IHC results to confirm the type of ARLGMD.

Sequence analysis of TCAP gene was undertaken in the eight cases which were found to be LGMD2G based on immunoblotting. All patients hailed from South India and shared the ethnic origin (Dravidian linguistic group). Family members, wherever available, were included in the analysis. Among these 7 families, one (F50) had two more affected siblings. Family (F111) also had one affected sibling (F111-2), who was available for clinical examination and genetic analysis, but muscle biopsy was not done. In total, we have studied 8 patients and 49 related family members. The inclusion criteria for the index cases were: clinical phenotype consistent with LGMD2G, elevated creatine kinase (CK) level, and loss or severe reduction of telethonin expression in IB studies. One hundred and sixty seven control samples, after ruling out muscular dystrophy or family history of muscular dystrophy, were recruited for comparison purpose.

### Histopathology and Immunohistochemistry (IHC)

#### Muscle biopsy

Open biopsy from the quadriceps femoris or biceps brachii muscle was performed in 280 patients. Samples were flash frozen in isopentane chilled in liquid nitrogen. Serial frozen sections were stained with hematoxylin and eosin (HE), modified gomoritrichome (MGT), succinic dehydrogenase (SDH), nicotinamide adenine dinucleotide (NADH-TR), succinic dehydrogenase–cytochrome C oxidase (SDH-COX), and adenosine triphosphatase (ATPase pH 9.4,4.6).

#### Antibodies

IHC staining was carried out on fresh frozen sections using monoclonal antibodies against dystrophin (1,2,3), sarcoglycans (α,β,δ,γ), dysferlin, caveolin-3, titin, merosin (α2 laminin) (Novocastra Laboratories, Newcastle, UK) and α- dystroglycan (Santacruz Biotechnology, Santacruz, CA, USA) as primary and HRP tagged NOVO linked as secondary antibody (Novocastra Laboratories, Newcastle, UK).

### Preparation of protein extracts, SDS-PAGE and immunoblot

Frozen muscle tissue (20 mg) was minced in 20 volumes of extraction buffer (75 mM Tris-HCl, pH 6.8, 15% SDS, 20% glycerol, 5% dithiothreitol and 0.001% bromophenol blue), homogenized and sonicated for 30 seconds (6 x 5s).The extract was boiled at 95°C for 5 minutesfollowed by centrifugation at 12000 rpm for 10 minutes. 10 µl of the supernatant was subjected to size fractionation using 15% SDS-PAGE followed by western blot using anti-Telethonin antibody (monoclonal antibody G-11, Santacruz Biotech) [21]. For myosin profile (loading control), protein extracts were run on 6% SDS-PAGE and stained with coomassie brilliant blue. To avoid artifactual protein degradation, which might reduce telethonin signal, precaution was taken to preserve the integrity of the biopsies and to maintain optimal protein loading and gel transfer conditions.

### Muscle MR Imaging

Muscle MRI was performed as a multi-sequence imaging protocol using a 1.5-T scanner (Magnetom, AERA, Siemens). Sections were obtained from axial planes including a T1-weighted spin-echo sequence (repetition time –918 msec, echo time 9.7 msec) and a short-time inversion recovery (STIR) sequence (repetition time 4020 msec, echo time 99 msec, inversion time 150 msec), in 10-mm slices. Involvement of all thigh and lower leg muscles was staged according to the intensity of the increased signals depicting fatty infiltration and STIR sequences for myoedema. Images were analyzed for signs of muscle degeneration, edema and normal/abnormal muscle bulk (hypertrophy, atrophy). Muscles evaluated on MRI: At *pelvis*: gluteus maximus, medius, minimus, piriformis, iliopsoas; *Thighs*: vastusmedialis, intermedius and lateralis, gracilis, sartorius, adductor muscles, tensor fasciae latae, rectus femoris, semimembranosus, semitendinosus, biceps femoris; *Lower legs*: tibialis anterior, extensor hallucis longus, peroneii, soleus, popliteus, extensor digitorum longus, medial and lateral head of gastrocnemius, tibialis posterior. The fibro-fatty replacement was evaluated on T1sequences by applying Mercuri score [22]; and myoedema was evaluated on STIR sequences by using a specific new oedema score [23].

### Genetic analysis of *TCAP*

About 5.0 ml of peripheral blood sample from each participant was collected in EDTA vacutainers. DNA was isolated according to the protocol described in our earlier study [24]. The coding region, including exon-intron boundaries and the UTR regions of Telethonin gene were amplified using 4 sets of primers. The sequence of primers and the conditions used for amplification are given in Table 1. PCR was performed in 0.2 ml thin-walled tubes using 50.0 ng of genomic DNA, 10 pmol of each primer, 200 µM of dNTPs, PCR buffer containing 1.5 mM MgCl_2_, and 2 units of AmpliTaqGold (Perkin Elmer). Amplified products were evaluated using 2% agarose gel electrophoresis. PCR products were purified by treating with exonuclease I and Shrimp alkaline phosphatase (Amersham, Piscataway, NJ) at 37°C and 80°C for 15 minutes each. Sequencing of PCR products was carried out using 100.0 ng (2.0 µl) of PCR product and 4 pmol (1.0 µl) of primer (forward and reverse in separate reactions), and 4.0 µl of BigDye Terminator ready mixture (Applied Biosystem, Foster city, USA). Cycle sequencing was carried out in a GeneAmp 9600 thermal cycler (Applied Biosystem, Foster city, USA) employing the following conditions: 30 cycles at 96°C for 10 seconds, 50°C for 5 seconds, and 60°C for 4 minutes. PCR amplicons were purified using the protocol described in our earlier study [25], dissolved in 10 µl of 50% Hi-Di formamide and analyzed using ABI 3700 automated DNA analyzer (Perkin Elmer). The sequences obtained were aligned with NCBI Reference Sequence: NG_008892.1 (gene)/NM_003673.3 (mRNA) using the AutoAssembler software to identify the sequence variations.

**Table 1:** Primer sequence used for the TCAP amplification.

Name	Forward	Reverse	Annealing temp
*TCAP* 1F	TTCTGCTTCCCACTTTATGAAAA	AGAAGGGAGAGTGCAACCAC	55
*TCAP* 2F	GTGAGGGTGACTGGGGACTA	GAAATTTCTCCAGGGCTTCA	54
*TCAP* 3F	GAGAGCAAAGGGGAACCAC	ACCCTCCCTTTGGGAACTC	55
*TCAP* 4F	GCCATGGCTGCTTTGTAGT	TGGGGTGTACCCATTAGCTC	54

## Results

### Clinical features

Based on the combined IHC and IB results, the commonest form of ARLGMD was LGMD 2B (Dysferlin) in 82/246 (33.33%); followed by 2I (FKRP) in 51/246 (20.73%), 2C-F (gamma, alpha, beta and delta sarcoglycan) in 35/246 (14.23%), 2A (calpain-3) in 25/246 (10.16%), and 2G (Telethonin) in 8/246 (3.25%). The remaining cases (45/246-18.30%) remained unclassified.

**Figure 1:**
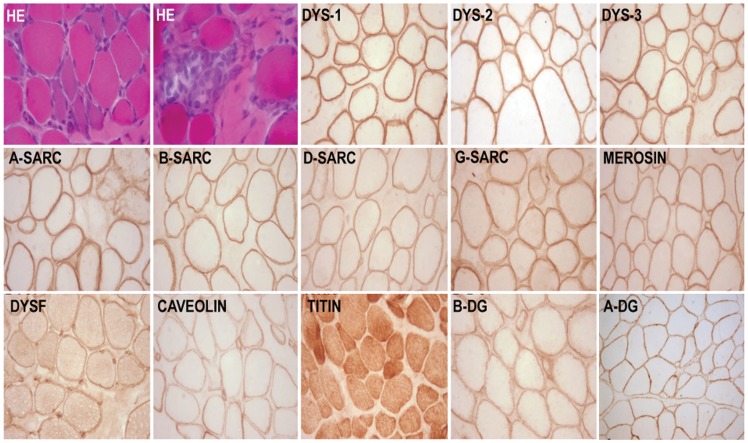
Transversely cut skeletal muscle tissue stained for Haematoxylin and eosin (HE) shows variation in fiber diameter clusters of basophilic regenerating fibers and myophagocytosis. Immunolabeling to antibodies against dystrophin (dys1,dys2,dys3) sarcoglycans (alpha-A, beta-B, delta-D, gamma-G, merosin, dysferlin (DYSF), caveolin, beta dystroglycan (B-DG), alpha dystroglycan (A-DG) shows uniform labeling along the sarcolemma and positive sarcoplasmic labeling to titin. X400

**Figure 2:**
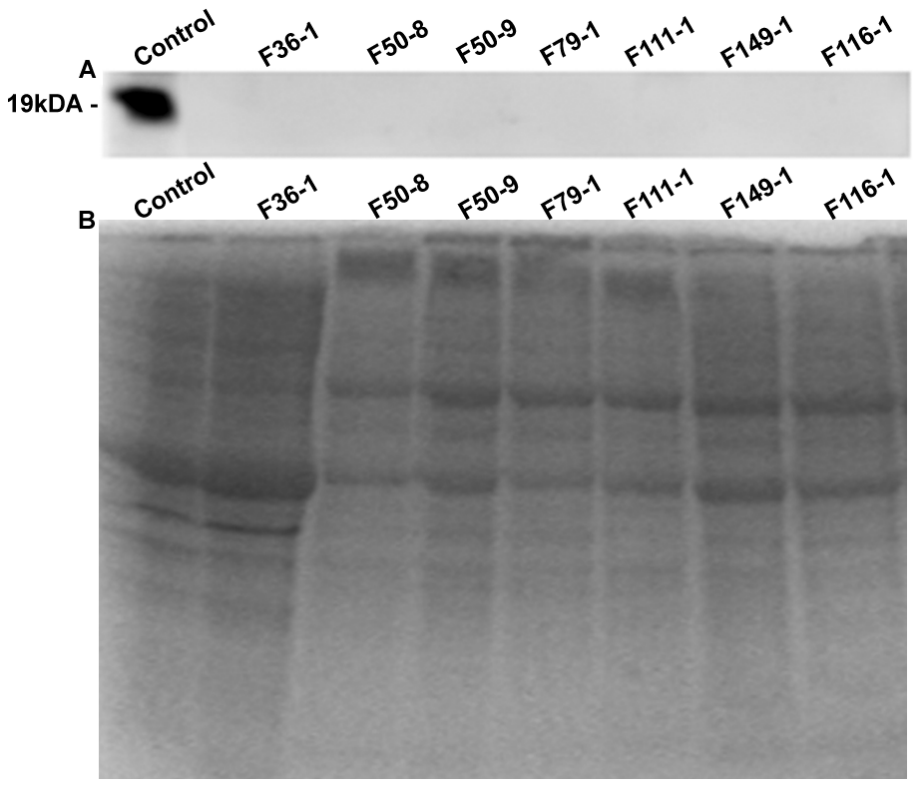
Western blot analysis of telethonin protein in muscle biopsies of control and telethoninopathy patients. **A**. Telethonin western Blot: Lane C corresponds to non-dystrophic positive control which shows telethonin band at 19 kDa. Lanes 1–7 corresponding to the Telethoninopathy patient samples, shows complete absence of the telethonin band. **B**. Coomassie stained SDS PAGE (15%) profile of total muscle extract from the samples.

The age of onset of LGMD2G cases ranged from 5 to 40 years (mean ±SD, 12.38±11.35) and the mean age at evaluation and mean duration of illness were 22.8±14.2, (9–52 years) and 8.50±6.87 (2–23 years), respectively. Gender distribution was equal. CK value ranged from 718 to 9253 IU/L (mean ± SD, 2574.4±2847.52). The clinical features and histopathological findings of the patients are presented in Table 2. IB studies in 7 patients (Biopsy not done for F111-2) showed normal expression of dystrophin, dysferlin, calpain-3 and all four sarcoglycans with complete or partial absence of telethonin on IB (Fig 1-2). A majority of patients had the typical phenotype with predominantly a proximo - distal form of involvement, scapular winging, muscle atrophy, presence of calf hypertrophy and foot drop and slowly progressive in most cases.

**Table 2:** Clinical features and Muscle Histopathology findings of patients with LGMD-2G.

Clinical features								
**Parameter/Patient ID**	F36-1	F50-1	F79-1	F97-1	F111-1	F111-2	F149-1	F116-1
**Age at presentation (Yrs)**	52	10	9	21	17	14	30	15
**Gender**	F	M	F	M	F	F	M	M
**Place of origin**	AP	KA	KA	TN	KE	KE	TN	KA
**Onset (Yrs)**	12	2	4	9	10	5	23	3
**Presenting symptom**	Difficulty in running fast; waddling	Difficulty in running fast	Slow in walking	Difficulty in running fast	Toe walking	Exertion induced muscle pain	Toe walking	Toe walking
**Hypertrophy**	absent	Calves	Triceps, calves	absent	Calves	Calves	absent	Absent
**Scapular winging**	Moderate	Moderate	Mild	Absent	Severe	Moderate	Mild	Severe
**Facial weakness**	absent	present	absent	absent	absent	absent	absent	Absent
**Contractures**	Ankle	Ankle	Ankle	Ankle, Hips	Ankle	Ankle	Ankle	Ankle
**Deltoids**	3-	5	4	4	4	4	4	4-
**Pectorals**	4	3-	4	4+	4	4	4	4+
**Biceps brachii**	4	4+	4	4	4	4	4	4-
**Triceps**	4	4-	3+	4	4	4	4	3+
**Distal UL**	5	5	5	5	5	5	5	5
**Glut. Medius**	3	3	2	3	2	3	1	5
**Hip adductors**	1	2	2	3+	1	1	1	2
**Iliopsoas**	3-	3	2	3+	1	3	1	3-
**Glut maximus**	1	2	2	3+	1	3	1	2
**Quadriceps**	3-	4	4+	3+	1	3	1	3-
**Hamstrings**	1	3+	3	5	1	3	3	3+
**Tibialis Anterior**	5	3+	4+	5	1	3	4+	4
**Gastrocnemius**	4	5	5	5	2	3+	4+	5
**CK (IU/L)**	1057	3729	1325	1583	719	9283	1281	1649
**MDFRS Scoring**	90	101	93	116	67	80	72	99
**Disability**	Dependent	Independent	Independent	Independent	Dependent	Independent	Dependent	Dependent

Muscle Histopathology findings								
**Fascicular architecture**	Severely effaced	Normal	Mild to moderately effaced	normal	Mild/moderate	-	Mild to moderate	Mild to moderate
**Myofibril shape and size**	Round fibers with marked variation	Round to Polygonal,/mild variation in size	Round/mild	polygonal	Round/mild variation	-	Round to polygonal/Mild variation	Round/marked variation
**Myophagocytosis/Necrotic fibers**	absent	absent	present/present	absent/absent	present/absent	-	absent/absent	Present
**Endomysial fibrosis/Adipose tissue infiltration**	Marked/marked	Mild to moderate/absent	Mild to moderate/absent	Absent/absent	Marked/marked	-	absent/mild to moderate	absent/mild to mod
**Regenerating fibers**	absent	absent	absent	absent	absent	-	present	Absent
**Hypertrophy**	absent	absent	present	absent	present	-	absent	Present
**Atrophy**	yes present	absent	present/present	present	present	-	absent	Present
**Splitting/internal nuclei**	present/occasional	present/occasional	present/occasional	absent/absent	present/occasional	-	present/occasional	present/present
**Inflammation**	absent	absent	absent	absent	absent	-	mild	Absent
**Lobulated fibers**	occasional	occasional	absent	occasional	absent	-	absent	Moth eaten fibers and lobulated fibers
**Ragged red and blue fibers**	present/absent	absent	absent	absent	absent	-	absent	Absent
**Rimmed vacuoles**	absent	absent	absent	absent	absent	-	absent	Absent

Yrs-Years; F-Female; M-Male; KA-Karnataka; TN-Tamilnadu; AP-Andhra Pradesh; EDB-Extensor digitorumbrevis; TA-Tibialis anterior; CK-Creatine kinase; MDFRS-Muscular dystrophy functional rating scale; Note: Biopsy was not done for patient F111-2 (sibling of patient F111-1).

### Muscle MRI findings

**Figure 3:**
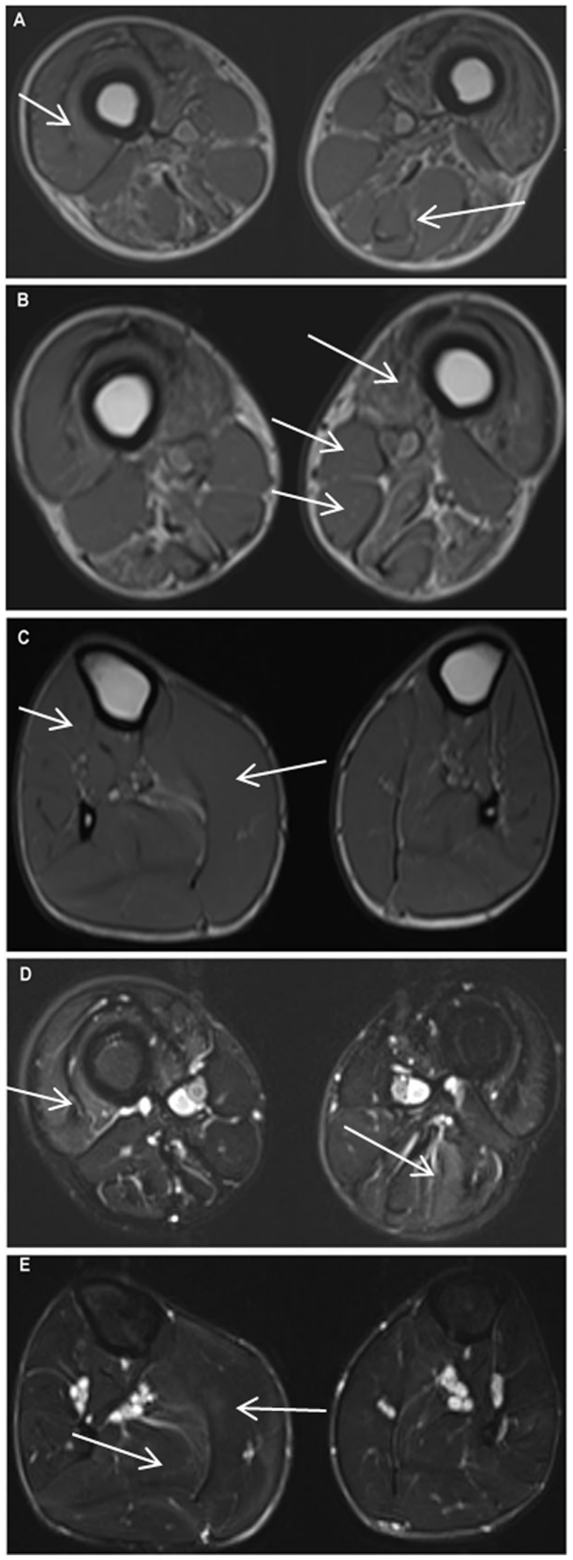
Muscle MRI of patient F97-1. **A**. T1W image showing global atrophy of muscles of anterior compartment, medial compartment and posterior compartment of thigh. Biceps femoris long head shows stage 3 fatty infiltration. Short head of biceps femoris, sartorius and gracilis are hypertrophied; **B**. shows severely atrophied vastus intermedius muscle, Mercurie stage 2b infiltration of vastus medialis muscle. Short head of biceps femoris, sartorius and gracilis are hypertrophied; **C**. Tibilialis anterior, extensor hallucis longus and extensor digitorum longus are atrophied (right more than left). On right side the medial head of gastrocnemius is hypertrophied and lateral head is atrophied. Left soleus is atrophied. Mild fatty infiltration of soleus muscle is noted in left side. Striking asymmetry is present; **D**. STIR image of thigh shows asymmetrical myoedema pattern. On the right side there is stage 3a myoedema and on the left side it is stage 2a. There is stage 2b myodema noted in the posterior compartment on left side; **E**. Myoedema mainly seen in gastrocnemius and soleus muscle. Stage 2b on right side and stage 2a on left side.

MRI findings of the patient, 97-1 (Fig 3), revealed: At Pelvis: gluteus maximus showed stage 2b fatty infiltration and iliopsoas showed stage 2a involvement. Rectus femorus was atrophied; At thighs: global atrophy of muscles of all three compartments. Sartorius, gracilis, semitendinosus and short head of biceps were spared and also hypertrophied. In the anterior compartment vastus, intermedius showed severe atrophy. Myoedema was mainly seen in the anterior compartment and more on the left side. In the posterior compartment, stage 2b myoedema was seen only on the left side; At legs: Atrophy of tibialis anterior, extensor digitorum longus, extensor hallucis longus, flexor hallucis longus, flexor digitorum longus was noted. Medial head of gastrocnemius was hypertrophied. Soleus appeared to be atrophied on left side and preserved on right side. Myoedema was noted only in gastrocnemius and soleus muscles. MRI staging of the affected individual muscles are listed in table 3.

**Table 3:** MRI staging of the individual muscles of lower limb for presence of fatty infiltration and myoedema.

Muscle	Mercurie staging		Myoedema staging		Atrophy		Hypertrophy	
	Right	Left	Right	Left	Right	Left	Right	Left
Gluteus maximus	2b	2b	0	0	Yes	Yes		
Iliopsoas	2a	2a	0	0	Yes	Yes		
Tensor facia lata	0	0	0	0	No	No		
Vastuslateralis	0	0	3a	0	Yes	Yes		
Vastusintermedius	1	1	3a	0	Yes	Yes		
Vastusmedialis	2b	2b	2a	0	Yes	Yes		
Rectus femoris	0	0	0	0	Yes	Yes		
Sartorius	0	0	0	0	No	No	Yes	Yes
Gracilis	0	0	0	0	No	No	Yes	Yes
Semimembranous	2b	2b	0	2b	Yes	Yes		
Semitendinosus	0	0	0	2b	No	No	Yes	Yes
Biceps long head	3	3	0	1	Yes	Yes		
Biceps short head	0	0	0	2b	No	No	Yes	Yes
Adductor brevis	1	1	0	0	Yes	Yes		
Adductor longus	1	1	0	0	Yes	Yes		
Adductor magnus	3	3	0	0	Yes	Yes		
Tibialis anterior	0	1	0	0	Yes	Yes		
Tibialis posterior	0	0	0	0	Yes	Yes		
Peroneus longus	0	0	0	1	No	No		
Soleus	0	1	2b	2a	Yes	Yes		
Gastrocnemius medialis	0	0	2b	2a	No	No	Yes	No
Gastrocnemius lateralis	0	0	2b	2a	Yes	Yes		
Extensor digitorumlongus	0	1	1	0	Yes	Yes		
Extensor hallucislongus	0	1	1	0	Yes	Yes		
Flexor digitorumlongus	0	1	1	1	yes	yes		
Flexor hallucislongus	0	1	1	1	yes			

### Sequence analysis

**Figure 4:**
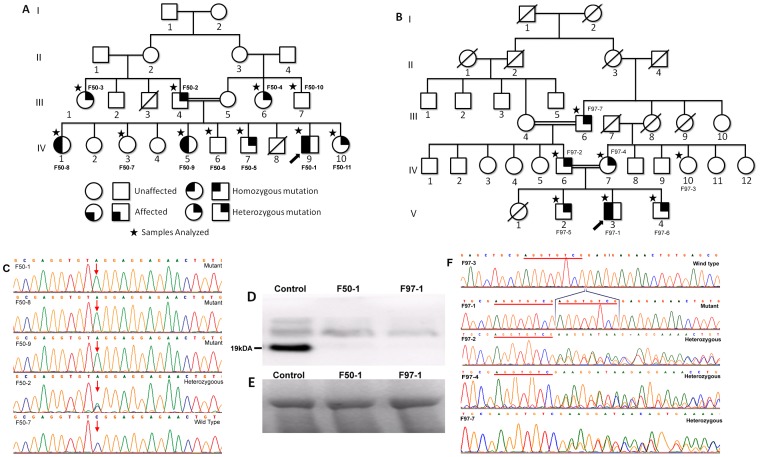
Mutational analysis of TCAP gene. A&B. Pedigree of F50 & F97 families respectively and the arrow indicates the proband; C. Electrophorogram representing heterozygous, homozygous and wild type c.32C>A mutation from family F50; Arrow indicates the polymorphic site; D. IB representing absence of telethonin band in F50-1 and F97-1; (E). Represents myosin bands of F50-1 and F97-1; (F). Electrophorogram representing homozygous, heterozygous and wild type c.26_33dupAGGTGTCG mutation in family F97.

The *TCAP* gene was analysed in ten patients (including two affected siblings from family F50), belonging to seven unrelated families. We have identified four different mutations that included; one novel and three reported variations (www.dmd.nl). Mutation numbering was based on *TCAP* cDNA with sequence position 1 being the A in the first ATG codon. The novel mutation (c.32C>A) (Fig 4C) is a nonsense substitution in the first exon, which introduces a stop codon (TCG to TAG) in three patients (homozygous mutant) of a consanguineous family (F50) (Table 4). All the unaffected family members were either heterozygous or homozygous for the alternate allele (Fig 4A). None of the control samples had this mutation.

**Table 4:** Description of genetic analysis of *TCAP* gene.

Patient ID	Nucleotide change	Protein change	State
F36-1	c.-178G>T	NA	Homozygous
	c.453A>C	p.( = )	Homozygous
F50-1	* **c.32C>A**	**p.(Ser11** * **)**	Homozygous
	c.-178G>T	**NA**	Heterozygous
	c.453A>C	p.( = )	Heterozygous
F50-8	* **c.32C>A**	**p.(Ser 11** * **)**	Homozygous
	c.-178G>T	**NA**	Homozygous
	c.453A>C	p.( = )	Homozygous
F50-9	* **c.32C>A**	**p.(Ser 11** * **)**	Homozygous
	c.-178G>T	NA	Homozygous
	c.453A>C	p.( = )	Homozygous
F79-1	c.-178G>T	NA	Homozygous
	c.453A>C	p.( = )	Homozygous
F97-1	**c.26 -33 Dup**	p. (Arg12fs31*)	Homozygous
	c.-178G>T	NA	Homozygous
	c.453A>C	p.( = )	Homozygous
F111-1	c.-178G>T	NA	Heterozygous
	c.453A>C	p.( = )	Heterozygous
F111-2	c.-178G>T	NA	Heterozygous
	c.453A>C	p.( = )	Heterozygous
F149-1	c.-178G>T	NA	Homozygous
	c.453A>C	p.( = )	Homozygous
F116-1	c.-178G>T	NA	Homozygous
	c.453A>C	p.( = )	Homozygous

*Novel mutation.

The second variation is an eight base pair duplication in the first exon (c.26_33dupAGGTGTCG), (Fig 4F) which leads to an amino acid change from 12th codon and terminates at 31stcodon, resulting in the formation of a truncated protein in one of the patients (F97-1) (Table 4). Both the parents, two brothers, and the grandfather were found to be heterozygous for this duplication and were unaffected (Fig 4B). This mutation was not found in the control samples. Two other mutations; c.453A>C; *p.( = )* in exon2and c.-178G>T of the 5′ UTR region; were polymorphic and found in all the patients, their relatives, and control samples.

## Discussion

For the first time in India, we have performed a comprehensive analysis of LGMD2G, a rare muscle disease caused by telethonin protein deficiency encoded by the *TCAP* gene. The clinical manifestations in all our patients was classical, with a phenotype consisting of mild to moderate limb girdle muscle weakness, significant thigh and anterior leg atrophy with weakness, mild scapular winging and calf hypertrophy. The mutational analysis revealed one novel and three reported substitutions.

The c.32C>A, p.(Ser11*) mutation is a novel, nonsense change and exists in the N-terminal region of the gene. Serine in the codon 11 was replaced by premature translational termination codon and it may lead to nonsense mediated decay or truncated protein. This mutation was found in a patient born to consanguineous parents (F50-1). The proband was a 10 year old boy and was the ninth child born to a first cousins marriage. He presented with exertion induced calf muscle pains and progressive toe walking from eight years of age, progressive pectoral girdle, arm and lower limb proximal muscle wasting with weakness, scapular winging and mild foot drop. There was significant global wasting of the thigh and anterior leg muscles with hypertrophied calves and tip toe walking. Biceps muscle biopsy revealed a dystrophic changes. He had two sisters (F50-8 and F50-9) with similar phenotype, both of who had this mutation in the homozygous condition (Fig 4A). The case F50-8, aged 25 years, attained wheelchair bound state at 21 years of age and the case F50-9 is ambulant at 17 years of age. All three affected individuals of a family had the same mutation, were affected by eight years of age and showed similar phenotype. The father, one sister, one brother, one paternal uncle, and a maternal aunt were heterozygous for this mutation and were unaffected. The other unaffected brother, sister, and maternal uncle did not have the mutation (Fig 4A). This clearly shows the recessive pattern of the disease and pathogenicity associated with the mutation c.32C>A.

**Figure 5:**
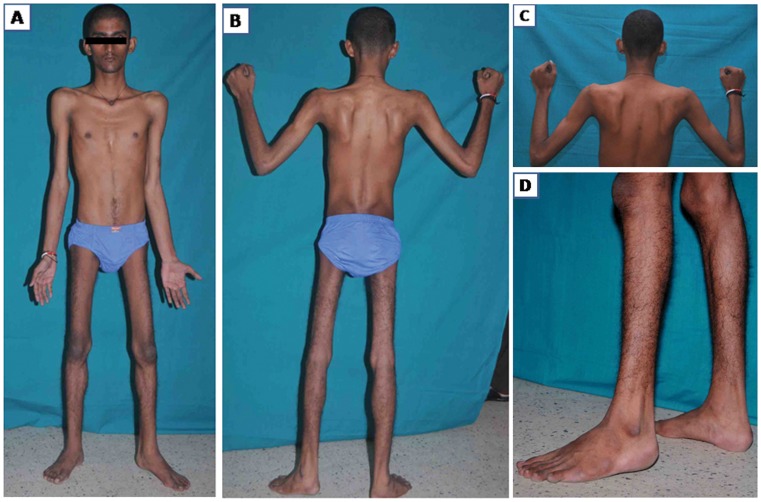
Clinical phenotype of patient F97-1. A. Slender habitus, B. Generalised poor muscle mass, global wasting of thigh muscles, C. moderate scapular winging, D. mild clf hypertrophy with minimal ankle contracture.

Frame shift variation c.26_33dupAGGTGTCG, (eight base pair duplication) in the first exon observed in a 21 year old proband (Fig 5) leads to an amino acid change from 12th codon and terminates the reaction at 31st codon, p.(Arg12fs31*). This may result in a truncated protein or nonsense mediated decay. The c.26_33dupAGGTGTCG has been reported in three unrelated Chinese families [26] and ours is the fourth family from Asia, but the first from India. This mutation is consistent with previous reports [3], [26] confirming that homozygous individuals have the typical LGMD2G phenotype. This patient (F97-1) presented with motor disabilities since early childhood with inability to run fast. By the age of 10 he had progressive generalized wasting and lower limb proximal muscle weakness. He was slender built, had moderate scapular winging, mild calf hypertrophy and contractures at the hips and ankles (Fig 5). There was MRC grade 4- weakness of the gluteus maximus, iliopsoas, quadriceps and hamstrings with mild asymmetry. Foot dorsiflexors were grade 3+ and other groups had normal power with elevated CK level and normal ECG and 2D ECHO. EMG was myopathic, nerve conductions were normal, pulmonary function tests showed moderate restrictive abnormality, and biceps muscle biopsy revealed dystrophic features. Parents, two brothers, and the grandfather were found to be heterozygous and unaffected.

Both, c.32C>A and c.26_33dupAGGTGTCG, mutations lead to truncation of the telethonin protein. Interestingly, all of the mutations reported (p.Gln35*, g.637–640del2, p.Trp25*, c.26_33dupAGGTGTCG), so far, in the *TCAP* gene in LGMD2G have been either nonsense or frame shift variation, which either gives rise to a truncated protein or terminates protein synthesis [2], [4], [27], [28]. To this list, we are adding a novel nonsense mutation.

Out of the eight patients studied, few had significant degree of toe walking from the onset of illness and this feature has been described earlier. [4]. In the previous reports, the onset of illness ranged between 2–15 years of age, with muscle weakness in all limbs, calf hypertrophy, and marked weakness of distal anterior leg muscles [29], [30]. Our patients also had similar age of onset and a comparable pattern of muscle involvement. Even though it is reported that *TCAP* is necessary for the cardiomyocyte's stretch sensor and the structural organization of the cardiac sarcomere [31], [32], [33], none of our cases had evidence of clinical or echocardiographic evidence of cardiac dysfunction at the time of clinical evaluation. Muscle biopsy has been reported to show dystrophic features with or without rimmed vacuoles [4], [27]. In our study, rimmed vacuoles were not noted in any case.

Certain muscle MRI findings observed in one of our proband's (case- 97-1) were comparable to those reported in a previous report [5]. Clinical examination revealed marked global atrophy of thigh muscles and atrophy of muscles in the anterior compartment of the leg. MRI showed a comparable pattern with atrophy of muscles in all three compartments of the thigh and anterior leg muscles. Sartorius, gracilis, semitendinosus, short head of biceps and medial head of gastrocnemius were spared and also hypertrophied. Anterior tibialis, extensor hallucis longus and extensor digitorum longus muscles showed prominent atrophy. The gastrocnemius muscles were slightly atrophic. Symmetry was noted in all the features. Only one previous report on a 50 year old Portuguese patient, describes the MRI findings of the muscles of the thighs and legs [5]. Myoedema was observed in anterior thigh and posterior leg muscles, which were relatively less affected. These features are unique and have not been reported so far. The Portuguese patient had demonstrated a homozygous nonsense c.157C>T (p.Gln53*) mutation in exon 2 of the TCAP gene, as described among Brazilian patients [1]. We have identified a 8 bp duplication c.26_33dupAGGTGTCG, which is reported among Chinese patients [26].Although LGMD2G may have phenotypic characteristics ofLGMD-2A, 2B, the sarcoglycanopathies and distal myopathies with rimmed vacuoles (DMRV), it could be well differentiated from these disorders noting that LGMD-2A usually does not have thigh atrophy or rimmed vacuoles. LGMD-2B has predominantly posterior leg muscle weakness with calf muscle wasting, sarcoglycanopathies do not have foot drop or rimmed vacuoles, DMRV have progressive anterior leg muscle wasting and weakness with sparing of quadriceps. In comparison, in LGMD2G there is presence of significant atrophy of the thigh muscles including quadriceps and significant involvement of anterior tibialis. The predominant involvement of pelvic girdle muscles with calf hypertrophy observed in LGMD2G resembles the phenotype of some LGMD-2I. However, in 2G patients the thigh muscles are severely affected along with tibialis anterior weakness and wasting [29], [30]. Thus, patients in the present series had the typical phenotypes described in LGMD2G and the genetic analysis confirms the same in two cases.

It has been reported that at least 25% of families and 40% of the isolated cases have no detectable mutation in any of the reported LGMD genes [34]. In agreement to this, we did not find any genetic variation in the telethonin gene in the remaining six IB confirmed cases. However, all of them showed partial or complete absence of telethonin on IB. This may be due to the secondary reduction of *TCAP* protein by some other Z-disk gene and probably involves a combination of genetic factors, since no protein in the Z-disc can be considered as an island separated from the others [35]. Many human diseases however, can be expected to be genetically heterogeneous caused by any one of several genes. The autosomal recessive limb-girdle muscular dystrophies represent a heterogeneous group of diseases which may be characterised by one or more autosomal loci. Further investigations may help in identification of additional genes responsible for LGMD2G.

In conclusion, we report LGMD2G cases for the first time from India. In this study, among the eight Indian LGMD2G patients, we found two telethonin mutations which include a novel c.32C>A mutation and a reported c.26_33dupAGGTGTCG duplication; both may be responsible for the formation of a truncated protein or nonsense mediated decay. Further investigation is needed to find the unknown factor for the telethonin deficiency in the remaining six cases.

## References

[1] Moreira ES, Wiltshire TJ, Faulkner G, Nilforoushan A, Vainzof M, et al (2000) Limb-girdle muscular dystrophy type 2G is caused by mutations in the gene encoding the sarcomeric protein telethonin. Nat Genet 24: 163–166. 10655062 10.1038/72822

[2] Moreira ES, Vainzof M, Marie SK, Sertie AL, Zatz M, et al (1997) The seventh form of autosomal recessive limb-girdle muscular dystrophy is mapped to 17q11-12. Am J Hum Genet 61: 151–159. 9245996 10.1086/513889 PMC1715843

[3] Yee W, Pramono Z, Tan C, Kathiravelu P, Lai P (2007) G.P.8.15 Limb girdle muscular dystrophy 2G and novel TCAP mutations in ethnic Chinese. Neuromuscular disorders: NMD 17: 814.

[4] Olive M, Shatunov A, Gonzalez L, Carmona O, Moreno D, et al (2008) Transcription-terminating mutation in telethonin causing autosomal recessive muscular dystrophy type 2G in a European patient. Neuromuscul Disord 18: 929–933. 18948002 10.1016/j.nmd.2008.07.009 PMC2592511

[5] Negrao L, Matos A, Geraldo A, Rebelo O (2010) Limb-girdle muscular dystrophy in a Portuguese patient caused by a mutation in the telethonin gene. Acta Myol 29: 21–24. 22029105 PMC2954583

[6] Valle G, Faulkner G, De Antoni A, Pacchioni B, Pallavicini A, et al (1997) Telethonin, a novel sarcomeric protein of heart and skeletal muscle. FEBS Lett 415: 163–168. 9350988 10.1016/s0014-5793(97)01108-3

[7] Mason P, Bayol S, Loughna PT (1999) The novel sarcomeric protein telethonin exhibits developmental and functional regulation. Biochem Biophys Res Commun 257: 699–703. 10208846 10.1006/bbrc.1999.0531

[8] McNally EM, Pytel P (2007) Muscle diseases: the muscular dystrophies. Annu Rev Pathol 2: 87–109. 18039094 10.1146/annurev.pathol.2.010506.091936

[9] Bertz M, Wilmanns M, Rief M (2009) The titin-telethonin complex is a directed, superstable molecular bond in the muscle Z-disk. Proc Natl Acad Sci U S A 106: 13307–133310. 19622741 10.1073/pnas.0902312106 PMC2726412

[10] Gao M, Lee E, Band AMR Case Study: Titin Ig domains.

[11] Knoll R, Buyandelger B (2013) Z-disc transcriptional coupling, sarcomeroptosis and mechanopoptosis. Cell Biochem Biophys 66: 65–71. 23065501 10.1007/s12013-012-9430-6 PMC3627051

[12] Lee EH, Gao M, Pinotsis N, Wilmanns M, Schulten K (2006) Mechanical strength of the titin Z1Z2-telethonin complex. Structure 14: 497–509. 16531234 10.1016/j.str.2005.12.005

[13] Knoll R, Hoshijima M, Hoffman HM, Person V, Lorenzen-Schmidt I, et al (2002) The cardiac mechanical stretch sensor machinery involves a Z disc complex that is defective in a subset of human dilated cardiomyopathy. Cell 111: 943–955. 12507422 10.1016/s0092-8674(02)01226-6

[14] Kojic S, Medeot E, Guccione E, Krmac H, Zara I, et al (2004) The Ankrd2 protein, a link between the sarcomere and the nucleus in skeletal muscle. J Mol Biol 339: 313–325. 15136035 10.1016/j.jmb.2004.03.071

[15] Nicholas G, Thomas M, Langley B, Somers W, Patel K, et al (2002) Titin-cap associates with, and regulates secretion of, Myostatin. J Cell Physiol 193: 120–131. 12209887 10.1002/jcp.10158

[16] Furukawa T, Ono Y, Tsuchiya H, Katayama Y, Bang ML, et al (2001) Specific interaction of the potassium channel beta-subunit minK with the sarcomeric protein T-cap suggests a T-tubule-myofibril linking system. J Mol Biol 313: 775–784. 11697903 10.1006/jmbi.2001.5053

[17] Haworth RS, Cuello F, Herron TJ, Franzen G, Kentish JC, et al (2004) Protein kinase D is a novel mediator of cardiac troponin I phosphorylation and regulates myofilament function. Circ Res 95: 1091–1099. 15514163 10.1161/01.RES.0000149299.34793.3c

[18] Tian LF, Li HY, Jin BF, Pan X, Man JH, et al (2006) MDM2 interacts with and downregulates a sarcomeric protein, TCAP. Biochem Biophys Res Commun 345: 355–361. 16678796 10.1016/j.bbrc.2006.04.108

[19] Frey N, Olson EN (2002) Calsarcin-3, a novel skeletal muscle-specific member of the calsarcin family, interacts with multiple Z-disc proteins. Journal of Biological Chemistry 277: 13998–14004. 11842093 10.1074/jbc.M200712200

[20] Markert CD, Meaney MP, Voelker KA, Grange RW, Dalley HW, et al (2010) Functional muscle analysis of the Tcap knockout mouse. Hum Mol Genet 19: 2268–2283. 20233748 10.1093/hmg/ddq105 PMC2865379

[21] Zardini E, Franciotta D, Melzi d'Eril GV (1993) Detection of dystrophin with a modified western blot technique in muscle tissue extracts. Clin Chem 39: 915. 8485897

[22] Mercuri E, Pichiecchio A, Allsop J, Messina S, Pane M, et al (2007) Muscle MRI in inherited neuromuscular disorders: past, present, and future. J Magn Reson Imaging 25: 433–440. 17260395 10.1002/jmri.20804

[23] Carlo B, Roberta P, Roberto S, Marina F, Corrado A (2006) Limb-girdle muscular dystrophies type 2A and 2B: clinical and radiological aspects. Basic Appl Myol 16: 17–25.

[24] Thangaraj K, Joshi MB, Reddy AG, Gupta NJ, Chakravarty B, et al (2002) CAG repeat expansion in the androgen receptor gene is not associated with male infertility in Indian populations. J Androl 23: 815–818. 12399527

[25] Thangaraj K, Ramana GV, Singh L (1999) Y-chromosome and mitochondrial DNA polymorphisms in Indian populations. Electrophoresis 20: 1743–1747. 10435442 10.1002/(SICI)1522-2683(19990101)20:8<1743::AID-ELPS1743>3.0.CO;2-V

[26] Waddell L, Lek M, Bahlo M, Bromhead C, Jones K, et al (2012) GP 41 The identification of LGMD2G (TCAP) in Australia. Neuromuscular Disorders 22: 831–832.

[27] Vainzof M, Moreira ES, Suzuki OT, Faulkner G, Valle G, et al (2002) Telethonin protein expression in neuromuscular disorders. Biochim Biophys Acta 1588: 33–40. 12379311 10.1016/s0925-4439(02)00113-8

[28] Waddell LB, Tran J, Zheng XF, Bonnemann CG, Hu Y, et al (2011) A study of FHL1, BAG3, MATR3, PTRF and TCAP in Australian muscular dystrophy patients. Neuromuscul Disord 21: 776–781. 21683594 10.1016/j.nmd.2011.05.007 PMC5210217

[29] Bonnemann C, Bushby K (2004) The limb-girdle muscular dystrophies. In: Engel AG; Franzini Armstrong A, editors Myology 3rd McGraw-Hill: p. 1077–1121.

[30] Fischer D, Walter MC, Kesper K, Petersen JA, Aurino S, et al (2005) Diagnostic value of muscle MRI in differentiating LGMD2I from other LGMDs. J Neurol 252: 538–547. 15726252 10.1007/s00415-005-0684-4

[31] Bos JM, Poley RN, Ny M, Tester DJ, Xu X, et al (2006) Genotype-phenotype relationships involving hypertrophic cardiomyopathy-associated mutations in titin, muscle LIM protein, and telethonin. Mol Genet Metab 88: 78–85. 16352453 10.1016/j.ymgme.2005.10.008 PMC2756511

[32] Hayashi T, Arimura T, Itoh-Satoh M, Ueda K, Hohda S, et al (2004) Tcap gene mutations in hypertrophic cardiomyopathy and dilated cardiomyopathy. J Am Coll Cardiol 44: 2192–2201. 15582318 10.1016/j.jacc.2004.08.058

[33] Zhang R, Yang J, Zhu J, Xu X (2009) Depletion of zebrafish Tcap leads to muscular dystrophy via disrupting sarcomere-membrane interaction, not sarcomere assembly. Hum Mol Genet 18: 4130–4140. 19679566 10.1093/hmg/ddp362 PMC2758143

[34] Blandin G, Marchand S, Charton K, Daniele N, Gicquel E, et al (2013) A human skeletal muscle interactome centered on proteins involved in muscular dystrophies: LGMD interactome. Skelet Muscle 3: 3. 23414517 10.1186/2044-5040-3-3 PMC3610214

[35] Faulkner G, Lanfranchi G, Valle G (2001) Telethonin and other new proteins of the Z-disc of skeletal muscle. IUBMB Life 51: 275–282. 11699871 10.1080/152165401317190761

